# The relationship between dietary micronutrients intake and cognition test performance among school‐aged children in government‐owned primary schools in Kumasi metropolis, Ghana

**DOI:** 10.1002/fsn3.1162

**Published:** 2019-08-09

**Authors:** Reginald Adjetey Annan, Charles Apprey, Odeafo Asamoah‐Boakye, Satoru Okonogi, Taro Yamauchi, Takeshi Sakurai

**Affiliations:** ^1^ Department of Biochemistry and Biotechnology, Faculty of Biosciences, College of Science Kwame Nkrumah University of Science and Technology Kumasi Ghana; ^2^ Department of Agricultural and Resource Economics, School of Agricultural and Life Sciences University of Tokyo Tokyo Japan; ^3^ Department of Health Sciences, School of Medicine Hokkaido University Sapporo Japan

**Keywords:** cognition tests, dietary intakes, micronutrients, school‐aged children

## Abstract

Nutrients are critical for optimal brain development, and good nutritional status is associated with cognitive development and improvement. The relationship between micronutrients intake and cognition in Ghanaian school‐aged children has not been studied. The study investigated dietary intakes of micronutrients and cognition test performance of school‐aged children. A cross‐sectional study was undertaken among 438 school children, aged 9–13 years from ten randomly selected basic schools in Kumasi, Ghana. Socio‐demographic data were obtained from a structured questionnaire. Dietary intakes of iron, zinc, vitamin B_6_, folate, vitamin B_12_, and vitamin A were determined from repeated 24‐hr dietary recall data from 351 children, while cognition test was performed using a Raven's Coloured Progressive Matrices (RCPM), a 36‐question test*.* Among 351 children, 156 (44.4%) had inadequate zinc intake, whereas 96 (27.4%) had inadequate iron intake. More than 1 in 2 children had inadequate vitamin A intake while 55.8% and 53.0% had inadequate vitamin B_12_ and folate intakes, respectively. More school‐aged boys (66.3%) than girls (46.8%) had inadequate vitamin B_12_ intake (*χ* ^2^ = 13.393, *p* < .001), while for iron, folate, vitamin B_6_, zinc, and vitamin A, the differences were not significant. Mean RCPM test score differed significantly between school type (*p* < .001), but did not differ between the different ages, and between children with adequate and inadequate iron, zinc, vitamin B_12,_ vitamin B_6_, and vitamin A intakes, except for folate intake (*p* = .050). Weak positive significant associations were observed between RCPM test score and zinc and folate intakes (*p* = .050)*.* Dietary micronutrient intakes were inadequate in majority of these children, which put them at risk of weakened immune system and poor health, but did not show significant associations with RCPM performance. Further studies using other forms of cognition tests may help confirm our findings, and provide the impetus for the necessary interventions.

## 1 INTRODUCTION

**Figure 1:**
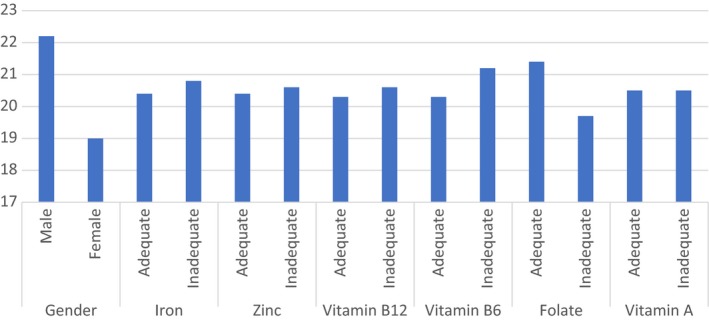
Mean cognitive test score between gender, and level of micronutrients intake. Comparison of mean cognitive score is all not significant, except for gender (*p* < .001) and folate intake (*p* = .05)

Proper brain function is a requirement for efficient cognitive function, and disruption of the brain reduces its efficiency (Bellisle, 2004). Good nutrition is necessary (Bryan, Osendarp, Hughes, & Baghurst, 2004), for brain function and cognitive performance, and thus should be adequately provided at all times (Bellisle, 2004). Poor nutritional status can indeed adversely affect brain function and cognitive performance (Bellisle, 2004). The proportion of children who do not obtain their full developmental potential is considerably large, and efforts to identify the specific causes of poor developmental outcomes are relevant (Peet et al., 2015). Micronutrient deficiencies are common among many developing countries, particularly among children, partly because of their higher physiological requirements and lower consumption of nutrient‐rich foods (Thankachan et al., 2013). The consumption of monotonous diets that are low in animal products and rich in phytates is the potential causes of deficiencies in iron, zinc, vitamin A, and vitamin B_12_ (Thankachan et al., 2013). New estimates show that nearly 250 million children under 5 years from low‐ and middle‐income countries are at risk of not reaching their developmental potential, partly due to poor nutrition during pregnancy and the first 2 years of life (Black et al., 2017). Therefore, poor micronutrients intake and nutrition in general, causing poor cognitive development, are among the reasons for many children not reaching their developmental potential (Figure 1).

Cognition is the ability to assimilate and process information received from different sources and to convert them into knowledge (Monti, Moulton, & Cohen, 2015). Cognitive processes include learning, attention, memory, language, reasoning, and decision‐making (Monti et al., 2015). Children especially of school age, require proper cognition for higher and excellent academic performance (Yehuda, Rabinovitzm, & Mostofskym, 2006). Most cognitive processes and activities are highly associated with brain function, physiology, and structure (Gomez‐Pinilla, 2008), and thus, its development may be affected by nutrition, physical activity, and social and economic status (Lemaire et al., 2010). During primary school education, deficiencies of vitamin A, iron, vitamin B_12_, vitamin B_6_, zinc, and folate among children can cause increased morbidity and negatively affect classroom attention and cognitive performance and thus devastate optimal schooling and academic performance (Fiorentino et al., 2017).

Vitamin B_12_ is an important micronutrient found only in animal food sources and is required for complete brain development and cognition (Rathod, Kale, & Joshi, 2016). Several studies conducted in developing countries have reported a high prevalence of vitamin B_12_ deficiency in children and attributed to inadequate vitamin B_12_ intake (Finkelstein, Layden, & Stover, 2015; Swaminathan, Thomas, & Kurpad, 2015; Venkatramanan, Armata, Struppm, & Finkelstein, 2016). Vitamin B_12_ deficiency is associated with poor cognitive development and growth in children (Strand et al., 2013; Moore et al., 2012; Van de Rest et al., 2012), and vitamin B_12_ status in infancy have been associated with development and performance on neuropsychological tests after 5 years (Kvestad et al., 2017).

Zinc deficiency may affect cognitive development by causing changes in neuropsychological behavior, and motor development, through its interactions with other nutrients (Black, 2003). Interventional studies on effects of zinc supplementation and cognitive performance in schoolchildren have produced conflicting results. Randomized controlled trials in Chinese and Mexican‐American children showed that zinc supplementation improved neuropsychological performance and reasoning, when compared with controls (Penland et al., 1999; Sanstead et al., 1998). In Canada, a randomized controlled trial found no significant effect of zinc supplementation on cognitive development in school‐aged children, compared with controls (Gibson et al., 1989).

Childhood iron deficiency anemia has been associated with delayed cognitive and motor development, and has negative effects on academic performance and educational fulfillment (Luo et al., 2017). In school‐aged children, cognitive function is impaired by iron deficiency with anemia (Grantham‐McGregor & Ani, 2001). A randomized cross‐over study by Sorensen et al. (2015) in Denmark found that school girls aged 8–11 years with few iron stores had a poorer overall “school performance” and poorer reading performance compared with girls with larger iron stores.

Vitamin A also regulates majority of the neurodevelopmental pathways that can plausibly influence cognition (Ali et al., 2017). A study by Buckley et al. (2013) in rural Nepal found that vitamin A supplementation from preconception through postpartum did not improve cognition development of children at 10–13 years. However, in Indonesia, provision of vitamin A supplement to infants showed slight improvement in motor development scores after 3 years (Humphrey et al., 1998).

The potential effect of adequate consumption of micronutrients on cognitive performance in school‐aged children is of special relevance. However, little is known regarding micronutrients intake and cognitive function in Ghanaian school children, though studies have shown high prevalence of micronutrients deficiencies in children. The above literature serves as backdrop for the aim of this study, which sought to assess the relationship between dietary intake of micronutrients and cognitive test performance of school‐aged children in Kumasi metropolis.

## 2 MATERIALS AND METHODS

### 2.1 Study design and participants

A cross‐sectional study design was adopted for this research. The study recruited 438 school‐aged children between ages 9 and 13 years, living and attending basic schools within the Kumasi metropolis. Participants were recruited from ten (10) randomly selected basic government schools in the Kumasi Metropolis. In each school, all school children within the required ages in primary five were recruited for the study.

### 2.2 Study area

The Kumasi Metropolis is one of the twenty‐seven (27) districts in the Ashanti region, with Kumasi as the district’s capital. The Kumasi Metropolis covers 254 square kilometers and encompasses 10 sub‐metropolitan areas: Manhyia, Tafo, Suame, Asokwa, Oforikrom, Asawasi, Bantama, Kwadaso, Nhyiaso, and Subin. It is located in South‐central Ghana. Kumasi metropolis is an important educational center with private‐owned and government‐owned primary and junior high schools, together referred to as basic schools. The metropolis has 203 government‐owned primary schools, and all were eligible for the study.

### 2.3 Sample size and sampling procedure

Statistically, 10 schools were determined appropriate for the study and each of the schools was randomly selected from all government‐owned primary schools in the Metropolis, such that each school had an equal chance of being selected. However, a convenience sample size of 500 was used for this study and 50 pupils were to be selected from each school. In each school, all children in primary 5, who were within the ages 9–13 years were chosen for the study. All children who were therefore present on the day of data collection were included in this study. Since some of the schools had less than 50 in the chosen class, the eventual number was less than 500. Sick and physically challenged pupils were excluded from the study, since anthropometrics and physical fitness tests had to be performed and those in this category were not fit for those procedures.

### 2.4 Data collection

Data were collected on their dietary micronutrients intake and cognition level. Socio‐demographic data such as age, gender, and socioeconomic status of parent/guardians were obtained. Data were collected by researchers and other trained field assistants. Data were collected between September 2016 and May 2017.

#### 2.4.1 Assessment of dietary intake

A 24‐hr triplicate recall on two weekdays and a weekend was used to collect dietary intakes of schoolchildren. Household food models of food items were used to identify quantities of foods eaten by participants. The weights (in grams) of foods consumed by participants were determined from the handy measures, and the composition of nutrients in meals was analyzed with the Nutrient Analysis Template, University of Ghana, Food Science, and Nutrition Department (2010). The dietary intakes of micronutrients (iron, zinc, vitamin B_6_, folate vitamin B_12,_ and vitamin A) were compared with Dietary Reference Intake (2000 and 2001) by Food and Nutrition Board, Institute of Medicine for recommended dietary allowances Zinc (2005 dietary reference; National Academy of Sciences & Food & Nutrition Board, Institute of Medicine, 2005). Some participants were absent at the time of collecting dietary intakes, so 351 participants reported their intakes.

#### 2.4.2 Cognition assessment test

The Raven’s Coloured Progressive Matrices (RCPM) test was used to assess the cognitive level of the schoolchildren. The cognition test was performed in a quiet environment, and the test procedures were explained to participants. The test contains three sets of twelve problems (36 colored questions), which measures fluid intelligence by problem‐solving and abstract reasoning by analogy, and has been used extensively as a culturally fair test of intelligence (Raven, 2000). The tests involved progressively geometrical designs and patterns with a missing piece, and each question has six to eight options to pick from and fill the missing piece. The cognitive tests were administered by well‐trained research assistants. The children were given a booklet containing the test and answer sheets to select the correct answer for each question. This was explained to the pupil prior to the test.

### 2.5 Data analysis

Data were analyzed using Statistical Package for Social Sciences version 25 (SPSS IBM Inc). Micronutrients intake values were converted into categories of adequacy or inadequacy. Data are expressed as the mean value ± standard deviation (*SD*) for continuous variables. Chi‐square cross tabulation was performed to find associations between adequate or inadequate micronutrient intake by gender and age group. The independent *t* test and ANOVA were used to compare mean cognition test scores by age group, gender, and micronutrients intake status and by school, while partial correlations (adjusted for dietary carbohydrate and protein intakes) were performed to test association between dietary micronutrients intake and cognition test scores. All tests were two‐tailed, and *p*‐values ≤.05 were considered statistically significant.

## 3 RESULTS

Table 1 presents socio‐demographic, average daily micronutrients intake of school‐aged children between 9 and 13 years. Among 438 study participants, 213 (48.6%) were males and 225 (51.4%) were females. Majority of the participants were at ages 11 (32.4%) and 12 (30.4%) years old. Majority of participants (61.3%, 55.8%, and 53.0%) had inadequate intake of vitamin A, vitamin B_12_, and folate, respectively. Also, of 351 children, 156 (44.4%) had inadequate zinc intake, whereas 96 (27.4%) had inadequate iron intake. Majority of the schoolchildren (63.8%) passed the percent RCPM test score, scoring above average percent (50%; Table 1) (Figure 1).

**Table 1:** Socio‐demographic, micronutrients intake and percent cognition test score of school‐age children (9–13 years)

Variable	Frequency	Percentage (%)
Gender	*N* = 438	
Male	213	48.6
Female	225	51.4
Age group (years)		
9	32	7.3
10	93	21.2
11	142	32.4
12	133	30.4
13	38	8.7
Micronutrients intake (Per day)	*N* = 351	
Dietary iron		
Adequate intake, 8 mg	255	72.6
Inadequate intake, less than 8 mg	96	27.4
Dietary zinc		
Adequate, 8 mg	195	55.6
Inadequate, less than 8 mg	156	44.4
Dietary vitamin B_12_		
Adequate, 1.8 µg	155	44.2
Inadequate, less than 1.8 µg	196	55.8
Dietary vitamin B_6_		
Adequate, 1.0 mg	276	78.6
Inadequate, less than 1.0 mg	75	21.4
Dietary folate		
Adequate, 300 µg	165	47.0
Inadequate, less than 300 µg	186	53.0
Dietary vitamin A		
Adequate, 2,000 IU	136	38.7
Inadequate, less than 2,000 IU	215	61.3
Percentage cognition test (%)		
Poor cognition, less than 50	127	36.2
Good cognition, 50–69.9	126	35.9
Excellent cognition, 70–100	98	27.9

Note

Based on Dietary Reference Intake (2005) by Food and Nutrition Board, Institute of Medicine.

Table 2 compares proportions of children who had adequate or inadequate micronutrients intake by gender and age. Proportion of male pupils with inadequate vitamin B_12_ intake was higher (66.3%) than female pupils (46.8%, *p* < .001). There were no significant differences between dietary micronutrients intake and age of participants. However, the 10‐year‐old pupils had the highest proportion of inadequate zinc (48.7%) and vitamin B_12_ (59.0%) intakes, whereas inadequate vitamin B_6_ (30.8%), folate (69.2%), and vitamin A (73.1%) were highest among the 9‐year‐old pupils. For dietary iron, vitamin B_6_, and folate, the female pupils (29.3%, 23.4%, 54.8%) had higher inadequate intakes compared with male pupils, respectively (25.2%, 19.0%, 50.9%), though none of the differences were statistically significant. Male pupils (64.4%) had a higher proportion of inadequate vitamin A intake compared with female pupils (58.5%, *p* = .273; Table 2).

**Table 2:** Distribution of micronutrients intake by gender and age of study population

Micronutrients intake (Per day)	Gender		*χ* ^2^	*p*‐Value	Age group (years) *N* (%)					*χ* ^2^	*p*‐Value
	Male, *N* (%) *N* = 163	Female, *N* (%) *N* = 188			9	10	11	12	13		
Dietary iron											
Adequate, 8 mg	122 (74.8)	133 (70.7)	0.739	.403^a^	19 (73.1)	59 (75.6)	82 (68.3)	75 (76.5)	20 (69.0)	2.420	.659^b^
Inadequate, less than 8 mg	41 (25.2)	55 (29.3)			7 (26.9)	19 (24.4)	38 (31.7)	23 (23.5)	9 (31.0)		
Dietary zinc											
Adequate, 8 mg	91 (55.8)	104 (55.3)	0.009	1.000^a^	15 (57.7)	40 (51.3)	68 (56.7)	55 (56.1)	17 (58.6)	0.808	.937^b^
Inadequate, less than 8 mg	72 (44.2)	84 (44.7)			11 (42.3)	38 (48.7)	52 (43.3)	43 (43.9)	43 (43.9)		
Dietary vitamin B_12_											
Adequate, 1.8 µg	55 (33.7)	100 (53.2)	13.393	<.001^a^	12 (46.2)	32 (41.0)	55 (45.8)	41 (41.8)	15 (51.7)	1.376	.848^b^
Inadequate, less than 1.8 µg	108 (66.3)	88 (46.8)			14 (53.8)	46 (59.0)	65 (54.2)	57 (58.2)	14 (48.3)		
Dietary vitamin B_6_											
Adequate, 1.0 mg	132 (81.0)	144 (76.6)	1.000	.193^a^	18 (69.2)	65 (83.3)	93 (77.5)	78 (79.6)	22 (75.9)	2.671	.614^b^
Inadequate, less than 1.0 mg	31 (19.0)	44 (23.4)			8 (30.8)	13 (16.7)	27 (22.5)	20 (20.4)	7 (24.1)		
Dietary folate											
Adequate, 300 µg	80 (49.1)	85 (45.2)	0.524	.269^a^	8 (30.8)	35 (44.9)	60 (50.0)	47 (48.0)	15 (51.7)	3.621	.460^b^
Inadequate, less than 300 µg	83 (50.9)	103 (54.8)			18 (69.2)	43 (55.1)	60 (50.0)	51 (52.0)	14 (48.3)		
Dietary vitamin A											
Adequate, 2,000 IU	58 (35.6)	78 (41.5)	1.283	.273^a^	7 (26.9)	39 (50.0)	46 (38.3)	35 (35.7)	9 (31.0)	6.808	.146^b^
Inadequate, less than 2,000 IU	105 (64.4)	110 (58.5)			19 (73.1)	39 (50.0)	74 (61.7)	63 (64.3)	20 (69.0)		

Note

Total = 351, *N*—frequency, %—percentage, *p*‐value is significant at *p* ≤ .05.

a Fisher's exact *p*‐values.

b chi‐square *p*‐values.

Table 3 presents proportions of children with adequate versus inadequate micronutrients intake by school type and also compares proportion of children with poor, good, or excellent cognition score by school type. The proportions with adequate or inadequate micronutrients intake vary by school type for all the micronutrients: dietary iron (*p* < .001), zinc (*p* = .001), vitamin B_12_ (*p* = .001), vitamin B_6_ (*p* = .025), folate (*p* = .017), and vitamin A (*p* = .001). Similarly, proportions of children with poor, good, or excellent cognition score varied by school type (*p* = .003).

**Table 3:** Distribution of micronutrients intake and cognition test by school type of study population

Variable micronutrients intake	School type *N* (%)										*p*‐Value
	School A	School B	School C	School D	School E	School F	School G	School H	School I	School J	
Dietary iron											
Adequate	48 (94.1)	19 (76.0)	33 (70.2)	24 (61.5)	27 (75.0)	12 (42.9)	37 (80.4)	21 (65.6)	14 (66.7)	20 (76.9)	<.001
Inadequate	3 (5.9)	6 (24.0)	14 (29.8)	15 (38.5)	9 (25.0)	16 (57.1)	9 (19.6)	11 (34.4)	7 (33.3)	6 (23.1)	
Dietary zinc											
Adequate	36 (70.6)	17 (68.0)	29 (61.7)	25 (64.1)	18 (50.0)	7 (25.0)	28 (60.9)	9 (28.1)	12 (57.1)	14 (53.8)	.001
Inadequate	15 (29.4)	8 (32.0)	18 (38.3)	14 (35.9)	18 (50.0)	21 (75.0)	18 (39.1)	23 (71.9)	9 (42.9)	12 (46.2)	
Dietary vitamin B_12_											
Adequate	28 (54.9)	13 (52.0)	18 (38.3)	18 (46.2)	23 (63.9)	4 (14.3)	24 (52.2)	7 (21.9)	7 (33.3)	13 (50.0)	.001
Inadequate	23 (45.1)	12 (48.0)	29 (61.7)	21 (53.8)	13 (36.1)	24 (85.7)	22 (47.8)	25 (78.1)	14 (66.7)	13 (50.0)	
Dietary vitamin B_6_											
Adequate	47 (92.2)	22 (88.0)	34 (72.3)	28 (71.8)	24 (66.7)	20 (71.4)	40 (87.0)	26 (81.3)	13 (61.9)	22 (84.6)	.025
Inadequate	4 (7.8)	3 (12.0)	13 (27.7)	11 (28.2)	12 (33.3)	8 (28.6)	6 (13.0)	6 (18.8)	8 (38.1)	4 (15.4)	
Dietary folate											
Adequate	36 (70.6)	7 (28.0)	24 (51.1)	16 (41.0)	16 (44.4)	10 (35.7)	24 (52.2)	11 (34.4)	9 (42.9)	12 (46.2)	.017
Inadequate	15 (29.4)	18 (72.0)	23 (48.9)	23 (59.0)	20 (55.6)	18 (64.3)	22 (47.8)	21 (65.6)	12 (57.1)	14 (53.8)	
Dietary vitamin A											
Adequate intake	30 (58.8)	9 (36.0)	19 (40.4)	12 (30.8)	12 (33.3)	3 (10.7)	25 (54.3)	14 (43.8)	4 (19.0)	8 (30.8)	.001
Inadequate intake	21 (41.2)	16 (64.0)	28 (59.6)	27 (69.2)	24 (66.7)	25 (89.3)	21 (45.7)	18 (56.3)	17 (81.0)	18 (69.2)	
Cognition test percent (%)											
Poor	11 (21.6)	15 (60.0)	13 (27.7)	15 (38.5)	18 (50.0)	5 (17.9)	9 (19.6)	17 (53.1)	10 (47.6)	14 (53.8)	.003
Good	20 (39.2)	5 (20.0)	21 (44.7)	16 (41.0)	9 (25.0)	12 (42.9)	19 (41.3)	10 (31.3)	8 (38.1)	6 (23.1)	
Excellent	20 (39.2)	5 (20.0)	13 (27.7)	8 (20.5)	9 (25.0)	11 (39.2)	18 (39.1)	5 (15.6)	3 (14.3)	6 (23.1)	

Note

Total = 351, *N*—frequency, %—percentage, poor—<50%, good—50%–69.9%, excellent—70%–100%, chi‐square *p*‐value is significant at *p* ≤ .05.

Table 4 presents cognition test outcome by gender, school type, and micronutrients intake. The average cognitive test score varied by gender (boys had a higher average) and school type (the highest score was 24.3 while the lowest was 16.3 points), and not by micronutrients intake (except for folate) and age. Children with adequate folate intake had 2.7 points in cognition score than those with inadequate folate intake (*p* = .050). However, mean cognition scores did not vary between children with adequate or inadequate intakes for iron, vitamin A, B12, B6 and zinc.

**Table 4:** Unadjusted mean comparison of cognition test scores by gender, school type, and micronutrients intake

Variables	Total number	Cognition Mean ± *SD* (*SEM*)	*p*‐value
	*N* = 351	RCPM score	
Total RCPM test score	351	20.6 ± 7.7 (0.4)^†^	
Gender			
Male	163	22.2 ± 7.6(0.6)^†^	<.001
Female	188	19.0 ± 7.8(0.6)	
Age group (years)			
9	26	18.9 ± 8.8 (1.7)^‡^	.231
10	78	19.7 ± 6.9 (0.8)	
11	120	21.8 ± 8.2 (0.7)	
12	98	19.9 ± 7.8 (0.8)	
13	29	20.5 ± 7.9 (1.5)	
School type			
School A	51	22.7 ± 5.9 (0.8)^a^ ^‡^	<.001
School B	25	18.4 ± 8.0 (1.6)	
School C	47	20.9 ± 6.7 (0.9)	
School D	39	19.4 ± 8.5 (1.4)	
School E	36	19.1 ± 9.0 (1.5)	
School F	28	24.3 ± 7.1 (1.3)^b^	
School G	46	23.8 ± 6.2 (0.9)^c^	
School H	32	16.8 ± 8.5 (1.5)^a,b,c^	
School I	21	18.3 ± 7.0 (1.5)	
School J	26	18.5 ± 7.7 (0.4)	
Micronutrients intake (Per day)			
Dietary iron			
Adequate	255	20.4 ± 7.8 (0.5)^†^	.666
Inadequate	96	20.8 ± 8.2 (0.8)	
Dietary zinc			
Adequate	195	20.4 ± 7.3 (0.5)^†^	.812
Inadequate	156	20.6 ± 8.5 (0.7)	
Dietary vitamin B_12_			
Adequate	155	20.3 ± 7.7 (0.6)^†^	.723
Inadequate	196	20.6 ± 8.1 (0.6)	
Dietary vitamin B_6_			
Adequate	276	20.3 ± 7.7 (0.5)^†^	.386
Inadequate	75	21.2 ± 8.4 (1.0)	
Dietary folate			
Adequate	165	21.4 ± 7.7 (0.6)^†^	.050
Inadequate	186	19.7 ± 8.0 (0.6)	
Dietary vitamin A			
Adequate intake	136	20.5 ± 7.9 (0.7)^†^	.965
Inadequate intake	215	20.5 ± 7.9 (0.5)	

Note

Data are presented as mean ± standard deviation (standard error mean), and *p*‐value is significant at *p* ≤ .05. Post hoc analysis showed significant mean differences between school types with same alphabets (a, *p*‐value = .016, b, *p*‐value = .004, c, *p*‐value = .002).

^†^ Independent *t* test.

^‡^ ANOVA.

In Table 5, we compare cognition scores by number of micronutrients that intakes were adequate for. Three groups were created, those with 0–2 nutrients adequate, those with 3–4 adequacy, and those with 5–6 nutrients adequate. Between these three groups, the mean cognition scores were not different (*p* = .753), although those with 5–6 nutrients adequate scored a slightly higher cognition score. Similar observations were showed when the children were grouped into those with 0–3 versus those with 4–6 nutrients adequate.

**Table 5:** Unadjusted mean comparison of combined adequacy for 6 micronutrients and cognition test score

Combined 6 micronutrients adequacy	*N* = 351	Cognition	*p*‐Value
		RCPM score	
Combined nutrients adequacy 1			
0–2 Nutrients adequacy	111	20.5 ± 8.3 (0.8)^a^	.753
3–4 Nutrients adequacy	121	20.1 ± 7.7 (0.7)	
5–6 Nutrients adequacy	115	20.9 ± 7.7 (0.7)	
Combined nutrient adequacy 2			
0–3 Nutrients adequacy	165	20.3 ± 8.1 (0.6)^b^	.745
4–6 Nutrients adequacy	186	20.6 ± 7.7 (0.6)	
Combined nutrients adequacy 3			
All nutrients inadequacy	46	19.9 ± 8.3 (1.2)	.970
All nutrients adequacy	52	20.4 ± 6.9 (0.9)	
1–5 nutrients adequacy	253	20.2 ± 8.1 (0.5)	

Note

Data are presented as mean ± standard deviation (standard error mean), and *p*‐value is significant at *p* ≤ .05.

a ANOVA.

b Independent *t* test analysis.

There was weak, nearly significant positive correlation between cognition test score and dietary zinc and folate intakes (*p* = .05). However, intakes of the other micronutrients had no significant association with cognition test scores (Table 6).

**Table 6:** Pearson's correlation between cognition test score and micronutrients intake

Micronutrients intake (Per day)	RCPM test score
	*r* (*p*‐Value)
Dietary iron	.097 (.080)
Dietary zinc	.106 (.050)
Dietary vitamin B_12_	.098 (.076)
Dietary vitamin B_6_	.096 (.082)
Dietary folate	.104 (.050)
Dietary vitamin A	.048 (.387)

Note

Adjusted for dietary carbohydrate and protein intakes, *p*‐value is significant at *p* ≤ .05 (two‐tailed).

## 4 DISCUSSION

The present study reports the relationship between dietary intake of micronutrients and cognition test performance of school‐aged children. There were more female participants than male participants indicating that gender disparity in school enrollment among children in the Metropolis is probably not an issue any more.

Micronutrient deficiencies in children from developing countries remain a public health issue as they are persistently common. Deficiencies of micronutrients can be attributed to inadequate dietary intake, low bioavailability of micronutrients, and anti‐nutritional inhibitors. Deficiencies in micronutrients increase risk of diseases and infection by weakening the immune system and further depleting nutrients stores (Katona and Katona‐Apte, 2008). Many studies have pointed out that deficiencies in vitamin A and iron are among the causes of anemia, infection, low immunity, morbidity, and child mortality (Beard, 2001; Ramakrishan, Aburto, McCabe, & Martorell, 2004). This study reported poor micronutrients intake among the schoolchildren, compared with their RDAs. Majority of participants (61.3%) had inadequate vitamin A intake while inadequate vitamin B_12_ (55.8%) and folate (53.0%) were reported in more than 5 in 10 schoolchildren. These imply that a large number of these children were likely to be micronutrients deficient at different degrees and their consequences, including poor health and neurodevelopmental outcomes. Previous studies in Ghana (Alicke et al., 2017; Egbi, Alatiah, Ayi, & Steiner‐Asiedu, 2017) had reported high prevalence of vitamin A deficiency (93.6%, 36.6%) in children aged 6–12 years. The story seems not to change. On the other hand, inadequate dietary iron intake was not as high in this study, with less than a third of the children having inadequate iron intakes. The inadequacies in micronutrients intake observed in this population are worrying as these children would soon be adolescents and their needs would be much higher. Therefore, interventions are needed to avert problems that may arise especially for the girl children as they step into puberty, especially with regards to iron deficiency.

There were no statistical differences between dietary micronutrients intake and the different ages. However, the 10‐year‐old schoolchildren recorded the highest proportion with inadequate zinc and vitamin B_12_, while the nine‐year‐olds had the highest proportions with vitamin B_6_, folate, and vitamin A, implying that the lower ages of this physiologic group are likely to have poorer intakes. Additionally, there were significant variations in micronutrients intake between the schools. The school with the highest proportions with adequate micronutrients intake showed close to the best cognition test scores compared with the other schools, implying that consumption of adequate micronutrients by school was related to better cognition test performance. However, a couple of schools that did not fall within the best of micronutrient intake also were recorded among the best cognitive performance. This suggests that micronutrients intake alone cannot be used to explain the cognition test performance.

Designed for children aged 5 through 13 years of age, the elderly, and mentally and physically impaired individuals, the RCPM test contains 36 progressively geometrical designs and patterns with a missing piece, listed in order of difficulty. It measures the test taker’s reasoning ability, meaning making, and general intelligence (Raven, 1936). Twelve questions each are in set A and B, and another 12 questions in set AB, inserted between A and B. Our study revealed that majority of the schoolchildren (63.8%) passed the percent RCPM test score, scoring above average percent (50%), and the overall mean RCPM test score was above average (20.6 ± 7.7). As no national data exist for this average to be compared, it is not clear whether the average score by the children in this study is a good average or not, apart from the fact that close to two‐thirds of the children scored over 50%.

Although in this study, the boys had a better RCPM test score than the girls, mean cognition test score did not differ by age, and whether the children had adequate or inadequate iron, zinc, vitamin B_6_, vitamin B_12,_ and vitamin A intake, except for folate. For folate, the children with adequate intake had a higher mean RCPM test score than those with inadequate intake, implying that folate intake, but not the other micronutrients, was related to the cognition test score. Evidence pointing to micronutrients intake and cognition is not consistent. A study by Boeke et al. (2013) in USA found no association between dietary vitamin B_12_ and cognitive outcomes. However, other studies, including Gewa et al., (2009) in Kenya and Ahmadi, Sohrabi, and Eftekhari (2009) in Iran, found that higher dietary vitamin B_12_ intake among schoolchildren was associated with improved cognitive outcomes. Clearly, further studies are required to elucidate these relationships, and as more studies on this subject are conducted, better understanding will be reached.

### 4.1 Limitations

The study assessed dietary intakes of participants but not serum levels, which may reflect the status of these nutrients better. It is also important to assess the overall patterns of intake rather than the specific nutrients. The dietary intakes were reported by schoolchildren directly, and therefore, over‐ or under‐estimation of portion sizes is possible. The study however incorporated the use of visual portion estimates like household handy measures during the data collection to help participants recall food portions consumed and reduce bias. The authors also recognize that the 24‐hr recall may not be the most reliable method for this age group. There was no comparison of income and other socioeconomic variables between the children, but it was expected that these children were from similar backgrounds by the fact that they were from government‐owned primary schools.

## 5 CONCLUSIONS

A large proportion of school‐aged children 9–13 years, attending government primary schools in Kumasi Metropolis, had inadequate dietary micronutrients intake (with the poorest intakes within ages 9 and 10) and therefore may be at risk of deficiencies and their devastating consequences, such as weakened immune system, poor health, increased risk for infectious diseases, and delayed physical and neurodevelopmental. This is the first in the population we studied that tried to find the relationship between micronutrients intake and RCPM scores, and although with the exception of folate, most of the nutrients were not significantly related to this particular cognition parameter, and further studies are needed using other cognition markers for a better understanding of this relationship. It may also be important to look at overall dietary pattern rather than specific nutrients for further studies. The findings however still call for interventions to promote adequate intake of nutrients, especially micronutrients in school‐aged children. Similar studies are recommended for children in private schools and/or other regions of Ghana.

## CONFLICT OF INTEREST

The authors declare that they do not have any conflict of interest regarding publication of this study.

## ETHICAL STATEMENT

Permission to carry out the study in the randomly selected schools was obtained from the Ghana Education Service (Kumasi Metropolis), following ethical approval from the Committee on Human Research Publication and Ethics (CHRPE) of the School of medical Sciences, KNUST. In each school, the study was explained to the Heads, who also gave approval and dates for data to be collected. All the children also gave informed assent to be included in the study, and parents/guardians were followed up for a household survey. No blood samples were taken from participants. We declare that this manuscript is an authentic product of our research and is not published or communicated for publication elsewhere either in part or full. The manuscript is an accurate account of the study being reported.

## References

[fsn31162-bib-0001] Ahmadi, A. , Sohrabi, Z. , & Eftekhari, M. H. (2009). Evaluating the relationship between breakfast pattern and short‐term memory in junior high school girls. Pakistan Journal of Biological Sciences, 12, 742–745. 10.3923/pjbs.2009.742.745 19634483

[fsn31162-bib-0002] Ali, H. , Hamadani, J. , Mehra, S. , Tofail, F. , Hasan, M. I. , Shaikh, S. , … Christian, P. (2017). Effect of maternal antenatal and newborn supplementation with vitamin A on cognitive development of school‐aged children in rural Bangladesh: A follow‐up of a placebo‐controlled, randomized trial. American Journal of Clinical Nutrition, 106, 77–87. 10.3945/ajcn.116.134478 28490513

[fsn31162-bib-0003] Alicke, M. , Boakye‐Appiah, J. K. , Abdul‐Jalil, I. , Henze, A. , van der Giet, M. , Schulze, M. B. , … Danquah, I. (2017). Adolescent health in rural Ghana: A cross‐sectional study on the co‐occurrence of infectious diseases, malnutrition and cardio‐metabolic risk factors. PLoS ONE, 12, e0180436 10.1371/journal.pone.0180436 28727775 PMC5519039

[fsn31162-bib-0004] Beard, J. L. (2001). Iron biology in immune function, muscle metabolism, and neuronal functioning. Journal of Nutrition, 131, 568S–579S. 10.1093/jn/131.2.568S 11160590

[fsn31162-bib-0005] Bellisle, F. (2004). Effects of diet on behaviour and cognition in children. British Journal of Nutrition, 92, S227–S232. 10.1079/BJN20041171 15522161

[fsn31162-bib-0006] Black, M. M. (2003). The evidence linking zinc deficiency with children’s cognitive and motor functioning. Journal of Nutrition, 133, 1473S–1476S. 12730446 10.1093/jn/133.5.1473S PMC3137935

[fsn31162-bib-0007] Black, M. M. , Walker, S. P. , Fernald, L. C. H. , Andersen, C. T. , DiGirolamo, A. M. , Lu, C. , … Grantham‐McGregor, S. (2017). Early childhood development coming of age: Science through the life course. Lancet, 389, 77–90. 10.1016/S0140-6736(16)31389-7 27717614 PMC5884058

[fsn31162-bib-0008] Boeke, C. E. , Gillman, M. W. , Hughes, M. D. , Rifas‐Shiman, S. L. , Villamor, E. , & Oken, E. (2013). Choline intake during pregnancy and child cognition at age 7 years. American Journal of Epidemiology, 177, 1338–1347. 10.1093/aje/kws395 23425631 PMC3676149

[fsn31162-bib-0009] Bryan, J. , Osendarp, S. , Hughes, D. , Baghurst, K. , et al. (2004). Nutrients for cognitive development in school children. Nutrition Reviews, 62, 295–306. 10.1301/nr.2004.aug.295-306 15478684

[fsn31162-bib-0010] Buckley, G. J. , Murray‐Kolb, L. E. , Khatry, S. K. , LeClerq, S. C. , Wu, L. , West, K. P. , & Christian, P. (2013). Cognitive and motor skills in school‐aged children following maternal vitamin A supplementation during pregnancy in rural Nepal: A follow‐up of a placebo‐controlled, randomised cohort. British Medical Journal Open, 3, e002000 10.1136/bmjopen-2012-002000 PMC3651971 23667158

[fsn31162-bib-0011] Egbi, G. , Alatiah, G. A. , Ayi, I. , & Steiner‐Asiedu, M. (2017). Red palm oil bean‐stew improved serum vitamin A and haemoglobin concentrations and anthropometric indicators of school children with low vitamin A concentrations in a malaria‐endemic setting. African Journal of Food, Agriculture, Nutrition and Development, 17, 12817–12836. 10.18697/ajfand.80.16750

[fsn31162-bib-0012] Finkelstein, J. L. , Layden, A. J. , & Stover, P. J. (2015). Vitamin B‐12 and perinatal health. Advances in Nutrition, 6, 552–563. 10.3945/an.115.008201 26374177 PMC4561829

[fsn31162-bib-0013] Fiorentino, M. , Perignon, M. , Kuong, K. , de Groot, R. , Parker, M. , Burja, K. , … Wieringa, F. T. (2017). Effect of multi‐micronutrient‐fortified rice on cognitive performance depends on premix composition and cognitive function tested: Results of an effectiveness study in Cambodian schoolchildren. Public Health Nutrition, 21, 816–827. 10.1017/S1368980017002774 29143707 PMC10261039

[fsn31162-bib-0014] Food Science and Nutrition Department, University of Ghana (2010). The nutrient analysis template software excel spreadsheet for Ghanaian foods.

[fsn31162-bib-0015] Gewa, C. A. , Weiss, R. E. , Bwibo, N. O. , Whaley, S. , Sigman, M. , Murphy, S. P. , … Neumann, C. G. (2009). Dietary micronutrients are associated with higher cognitive function gains among primary school children in rural Kenya. British Journal of Nutrition, 101, 1378–1387. 10.1017/S0007114508066804 18826659

[fsn31162-bib-0016] Gibson, R. S. , Vanderkooy, P. D. S. , MacDonald, A. C. , Goldman, A. , Ryan, B. A. , & Berry, M. (1989). A growth‐limiting, mild zinc‐deficiency syndrome in some Southern Ontario boys with low height percentiles. American Journal of Clinical Nutrition, 49, 1266–1273. 10.1093/ajcn/49.6.1266 2729165

[fsn31162-bib-0017] Gomez‐Pinilla, F. (2008). Brain foods: The effects of nutrients on brain function. Nature Reviews Neuroscience, 9, 568–578. 10.1038/nrn2421 18568016 PMC2805706

[fsn31162-bib-0018] Grantham‐McGregor, S. , & Ani, C. (2001). A review of studies on the effect of iron deficiency on cognitive development in children. Journal of Nutrition, 131, 649S–666S. 10.1093/jn/131.2.649S 11160596

[fsn31162-bib-0019] Humphrey, J. H. , Agoestina, T. , Juliana, A. , Septiana, S. , Widjaja, H. , Cerreto, M. C. , … West, K. P. (1998). Neonatal vitamin A supplementation: Effect on development and growth at 3 y of age. American Journal of Clinical Nutrition, 68, 109–117. 10.1093/ajcn/68.1.109 9665104

[fsn31162-bib-0020] Katona, P. , & Katona-Apte, J. (2008). The Interaction between Nutrition and Infection. Clinical Infectious Diseases, 46(10), 1582–1588. 10.1086/587658. 18419494

[fsn31162-bib-0021] Kvestad, I. , Hysing, M. , Shrestha, M. , Ulak, M. , Thorne‐Lyman, A. L. , Henjum, S. , … Strand, T. A. (2017). Vitamin B‐12 status in infancy is positively associated with development and cognitive functioning 5 y later in Nepalese children. American Journal of Clinical Nutrition, 105, 1122–1131. 10.3945/ajcn.116.144931 28330909

[fsn31162-bib-0022] Lemaire, J. B. , Wallace, J. E. , Dinsmore, K. , Lewinm, A. M. , Ghali, W. A. , & Roberts, D. (2010). Physician nutrition and cognition during work hours: Effect of a nutrition‐based intervention. BMC Health Services Research, 10, 241 10.1186/1472-6963-10-241 20712911 PMC2929232

[fsn31162-bib-0023] Luo, R. , Yue, A. I. , Zhou, H. , Shi, Y. , Zhang, L. , Martorell, R. , … Sylvia, S. (2017). The effect of a micronutrient powder home fortification program on anemia and cognitive outcomes among young children in rural China: A cluster randomized trial. BMC Public Health, 17, 38 10.1186/s12889-017-4755-0 28946866 PMC5613507

[fsn31162-bib-0024] Monti, J. M. , Moulton, C. J. , & Cohen, N. J. (2015). The role of nutrition on cognition and brain health in ageing: A targeted approach. Nutrition Research Reviews, 28, 167–180. 10.1017/S0954422415000141 26650244

[fsn31162-bib-0025] Moore, E. , Mander, A. , Ames, D. , Carne, R. , Sanders, K. , & Watters, D. (2012). Cognitive impairment and vitamin B12: A review. International Psychogeriatrics, 24, 541–556. 10.1017/S1041610211002511 22221769

[fsn31162-bib-0026] National Academy of Sciences, Food and Nutrition Board, Institute of Medicine (2005). Dietary reference intakes series. Washington, DC: National Academies Press. in text ‘Mahan, L.K., Escott‐Stump, S. and Raymond, J.L. (2012). Krause’s Food and the Nutrition Care Process, 13th Editions, Elsevier Saunders Publication, Missouri, USA, page 1.

[fsn31162-bib-0027] Peet, E. D. , McCoy, D. C. , Danaei, G. , Ezzati, M. , Fawzi, W. , Jarvelin, M. R. , … Fink, G. (2015). Early childhood development and schooling attainment: Longitudinal evidence from British, Finnish and Philippine birth cohorts. PLoS ONE, 10, e0137219 10.1371/journal.pone.0137219 26352937 PMC4564180

[fsn31162-bib-0028] Penland, J. , Sanstead, H. , Egger, N. , Dayal, H. , Alcock, N. , Plotkin, R. , … Zavaleta, A. (1999). Zinc, iron and micronutrient supplementation effects on cognitive and psychomotor function of Mexican‐American school children. The FASEB Journal, 13, 921.

[fsn31162-bib-0029] Ramakrishan, U. , Aburto, N. , McCabe, G. , & Martorell, R. (2004). Multimicronutrient interventions but not vitamin A or iron interventions alone improve child growth: Results from three meta‐analyses. Journal of Nutrition, 134, 2592–2602. 15465753 10.1093/jn/134.10.2592

[fsn31162-bib-0030] Rathod, R. , Kale, A. , & Joshi, S. (2016). Novel insights into the effect of vitamin B12 and omega‐3 fatty acids on brain function. Journal of Biomedical Science, 23, 17 10.1186/s12929-016-0241-8 26809263 PMC4727338

[fsn31162-bib-0031] Raven, J. C. (1936). Mental tests used in genetic studies: The performance of related individuals on tests mainly educative and mainly reproductive. MSc thesis, University of London.

[fsn31162-bib-0032] Raven, J. (2000). The Raven’s progressive matrices: Change and stability over culture and time. Cognitive Psychology, 41, 1–48. 10.1006/cogp.1999.0735 10945921

[fsn31162-bib-0033] Sanstead, H. H. , Penland, J. G. , Alcock, N. W. , Dayal, H. H. , Chen, X. C. , Li, J. S. , … Yang, J. J. (1998). Effects of repletion with zinc and other micronutrients on neuropsychologic performance and growth of Chinese children. American Journal of Clinical Nutrition, 68, 470S–475S. 9701162 10.1093/ajcn/68.2.470S

[fsn31162-bib-0034] Sorensen, L. B. , Damsgaard, C. T. , Dalskov, S. M. , Petersen, R. A. , Egelund, N. , Dyssegaard, C. B. , … Lauritzen, L. (2015). Diet‐induced changes in iron and n‐3 fatty acid status and associations with cognitive performance in 8–11‐year‐old Danish children: Secondary analyses of the Optimal Well‐Being, Development and Health for Danish Children through a Healthy New Nordic Diet School Meal Study. British Journal of Nutrition, 114, 1623–1637. 26359192 10.1017/S0007114515003323

[fsn31162-bib-0035] Strand, T. A. , Taneja, S. , Ueland, P. M. , Refsum, H. , Bahl, R. , Schneedem, J. , … Bhandari, N. (2013). Cobalamin and folate status predict mental development scores in North Indian children 12–18 months of age. American Journal of Clinical Nutrition, 97, 310–317. 23283502 10.3945/ajcn.111.032268

[fsn31162-bib-0036] Swaminathan, S. , Thomas, T. , & Kurpad, A. V. (2015). B‐vitamin interventions for women and children in low‐income populations. Current Opinion in Clinical Nutrition and Metabolic Care, 18, 295–306. 10.1097/MCO.0000000000000166 25807352

[fsn31162-bib-0037] Thankachan, P. , Selvam, S. , Surendran, D. , Chellan, S. , Pauline, M. , Abrams, S. A. , & Kurpad, A. V. (2013). Efficacy of a multi micronutrient‐fortified drink in improving iron and micronutrient status among schoolchildren with low iron stores in India: A randomised, double‐masked placebo‐controlled trial. European Journal of Clinical Nutrition, 67, 36–41. 10.1038/ejcn.2012.188 23232585

[fsn31162-bib-0038] Van de Rest, O. , van Hooijdonk, L. W. , Doets, E. , Schiepers, O. J. , Eilander, A. , & de Groot, L. C. (2012). B vitamins and n‐3 fatty acids for brain development and function: Review of human studies. Annals of Nutrition and Metabolism, 60, 272–292. 10.1159/000337945 22678093

[fsn31162-bib-0039] Venkatramanan, S. , Armata, I. E. , Struppm, B. , & Finkelstein, J. L. (2016). Vitamin B_12_ and cognition in children. Advances in Nutrition, 7, 879–888. 10.3945/an.115.01202 27633104 PMC5015033

[fsn31162-bib-0040] Yehuda, S. , Rabinovitzm, S. , & Mostofskym, D. I. (2006). Nutritional deficiencies in learning and cognition. Journal of Pediatric Gastroenterology and Nutrition, 43, S22–S25. 10.1097/01.mpg.0000255847.77034.a4 17204975

